# Association of chronic somatic multimorbidites with the sleep quality in patients with mild to moderate dementia; A cross-sectional study

**DOI:** 10.1192/j.eurpsy.2023.1224

**Published:** 2023-07-19

**Authors:** K. Bosak, I. Filipčić, I. Šimunović Filipčić, P. Folnegović Grošić, Ž. Bajić, V. Grošić

**Affiliations:** 1 Psychiatric Clinic Sveti Ivan, Zagreb; 2 Faculty of Dental Medicine and Health, Josip Juraj Strossmayer University of Osijek, Osijek; 3 School of Medicine, University of Zagreb; 4 Department of psychiatry and psychological medicine, University Hospital Center Zagreb; 5 Department of Psychiatry, University Hospital Centre Zagreb, Zagreb, Croatia

## Abstract

**Introduction:**

As much as one fifth of hospitalized patients with dementia may have sleep disturbance which may impair daily functioning, cause lower quality of life, chronic stress and consequently even deterioration of physical health. We hypothesized that multimorbidity may be one of many causes of higher incidence of sleep disturbances in hospitalized patients with dementia. Multimorbidity may be defined as having two or more chronic medical conditions. In 2018, the U.S. Academy of Medical Sciences declared studies on multimorbidity a global priority, noting the lack of research and the clinical importance and complexity of the problem.

**Objectives:**

To assess the association of chronic physical multimorbidity (CPM) with the quality of sleep, in population of hospitalized patients with dementia.

**Methods:**

We performed a cross-sectional study at University Psychiatric Hospital “Sveti Ivan”, Zagreb, Croatia. We selected a consecutive sample of patients diagnosed with mild or moderate dementia. The outcome was quality of sleep assessed by the Pittsburgh Sleep Quality Index. We recoded the number of chronic medical conditions from the hospital electronic health records, and defined multimorbidity as ≥ 2.

**Results:**

After the adjustment for possible confounders: age, gender, duration of hospitalization, dementia severity and treatment, patients having a CPM had 2.5 times higher odds for sleep disturbance than patients with no, or with only one chronic medical condition (OR=2.51; 95% CI 0.60-4.41; p=0.011). This association between CPM and sleep disturbance was markedly stronger in women (OR=3.42; 95% CI 0.40-6.45; p=0.029) than in men (OR=1.73; 95% CI -0.66-4.14; p=0.145). In patients with three, and four chronic medical conditions the odds for sleep disturbance growth rapidly (OR=3.65; 95% CI 0.53-6.77; p=0.023; OR=4.71; 95% CI 1.40-8.03; p=0.007, respectively).

**Conclusions:**

Chronic multimorbidity is associated with sleep disturbance in patients with mild or moderate dementia.

**Disclosure of Interest:**

None Declared

